# Physicians’ Perspectives on the Implementation of the Second Opinion Directive in Germany—An Exploratory Sequential Mixed-Methods Study

**DOI:** 10.3390/ijerph19127426

**Published:** 2022-06-17

**Authors:** Susann May, Dunja Bruch, Felix Muehlensiepen, Yuriy Ignatyev, Edmund Neugebauer, Cecile Ronckers, Sebastian von Peter

**Affiliations:** 1 Center for Health Services Research, Brandenburg Medical School (Theodor Fontane), 15562 Rüdersdorf, Germany; dunja.bruch@mhb-fontane.de (D.B.); felix.muehlensiepen@mhb-fontane.de (F.M.); yuriy.ignatyev@mhb-fontane.de (Y.I.); edmund.neugebauer@mhb-fontane.de (E.N.); 2 Brandenburg Medical School (Theodor Fontane), 16816 Neuruppin, Germany; cecile.ronckers@uni-oldenburg.de (C.R.); sebastian.vonpeter@mhb-fontane.de (S.v.P.); 3 Faculty for Health Sciences Brandenburg, Brandenburg Medical School (Theodor Fontane), 16816 Neuruppin, Germany; 4 Department of Health Services Research, Carl Von Ossietzky Universität Oldenburg, 26129 Oldenburg, Germany

**Keywords:** second opinion, surgery, patient safety, mixed-methods, informed decision making, health system

## Abstract

A new Second Opinion Directive (SOD) was introduced in Germany in December 2018 for hysterectomy, tonsillotomy, and tonsillectomy to support shared decision making and to avoid unnecessary surgeries. Owing to its recent implementation, evidence and insights regarding outcomes and challenges encountered with the SOD are lacking, notably from the physicians’ perspective. To assess this, we undertook an exploratory sequential mixed-methods design with an initial qualitative phase followed by a quantitative evaluation. A qualitative analysis of 22 interviews with specialists in gynecology and otorhinolaryngology was followed by a statistical analysis of a survey of 136 physicians in those disciplines. The specialists expressed a generally positive opinion of the new SOD, emphasizing the aspects of patient orientation, support in decision making, and patient safety. However, they also highlighted the following structural problems regarding the SOD implementation: In addition to an increased organisational effort, the specialists criticised the SOD with regard to its implementation in rural regions with a low availability of specialists for referral. Barriers that impede the implementation of the current directive, such as the adaptation of the qualifying requirements for authorized second opinion physicians, as well as the inclusion of relevant indications, need focused consideration to obtain better alignment with everyday practice.

## 1. Introduction

**Figure 1:**
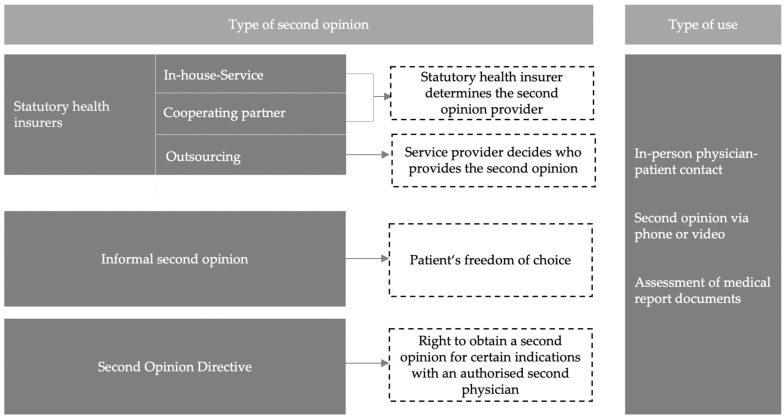
Options for obtaining a second opinion in Germany in accordance with Pieper [5].

Patients are becoming increasingly involved in decision making regarding their personal health care. This trend is in accordance with growing needs for knowledge as ever more patients are actively seeking information about their disease and treatment options, and are also searching for a second opinion [1]. Certainly, second opinions can support patients in their decision-making process in the face of uncertainty [2,3], which may help to avoid unnecessary medical testing and/or interventions [4]. The German health care system accommodates various forms of second opinions, which are summarized in Figure 1.

**Figure 2:**
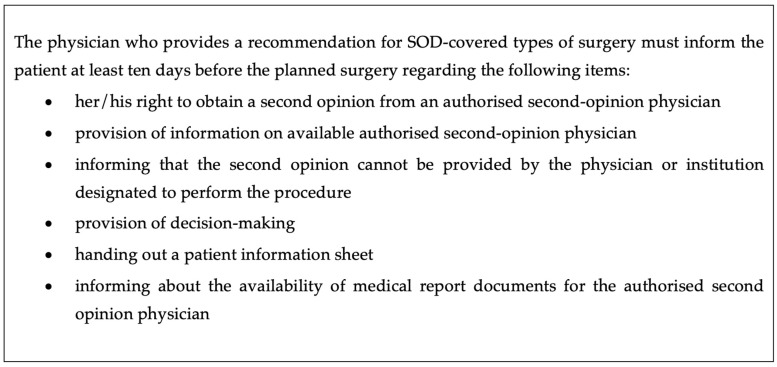
Mandatory Information obligations for the physician who first suggests surgery according to the 2018 Second Opinion Directive (SOD); decision-making aids are available at: https://www.iqwig.de/presse/pressemitteilungen/pressemitteilungen-detailseite_10140.html (assessed on 3 March 2022).

A wide variety of second opinion services are offered as part of health insurance packages. For patients with malignant tumours, consultation with a tumour board can constitute a second opinion. First and foremost, patients can consult other physicians at any time, and this kind of informal second opinion consultation is usually covered by health insurance. As of 1 December 2018, a new form of second opinion regulation was introduced in Germany with the Second Opinion Directive (SOD). Under the SOD, patients enrolled in statutory health insurance are entitled to obtain an independent and cost-free second opinion in the specific contexts of an indication for hysterectomy, tonsillectomy, or tonsillotomy [6]. Other indications, such as shoulder arthroscopy, implantation knee endoprosthesis, amputation for diabetic foot syndrome, and spinal surgery were subsequently included in the SOD during the course of the present project, which aims to assess the current status and effectiveness of the new directive. The SOD was introduced to support shared decision making and to avoid unnecessary surgeries for common indications [6]. The main selection criteria for the surgical indications regulated by SOD concern the volume of performed surgeries and regional practice variations across Germany, as well as international experience [7]. A physician who recommends one of the selected surgeries must adhere to a set of patient information requirements, which are listed in Figure 2. In Germany, diagnostic procedures and surgery recommendations are predominantly made in the outpatient setting.

Patients can seek a second opinion from an authorized second opinion physician. The qualification requirements are a specialist designation, at least five years of full-time practice and a post-doctoral lecture qualification (in German: Habilitation) or an examiner certification. The authorized second opinion physician must verbally inform the patient about the recommended surgery, and discuss possible alternative therapies or options for treatment. The authorized second opinion physician may provide a second opinion based on medical report documents, or may carry out further examinations if deemed necessary [6]. Owing to the recency of SOD-implementation, evidence and insights regarding its outcomes and the challenges encountered in its practice are lacking. Aside from patient perceptions and experiences, physician acceptance may be particularly relevant for outcome assessment; while SOD development is supervised by the Federal Joint Committee (in German: Gemeinsamer Bundesausschuss, GBA), practicing physicians carry ultimate responsibility for (and burden of) the adequate implementation of procedures and the application of the directive, which represents a decisive step towards improving patient care on a daily basis [8]. In general, studies on the introduction of new guidelines into medical routines reported low success rates, in particular when barriers and further contextual factors were not considered during the implementation phase [9]. In this study, we aim to elucidate the impact and proposed potential improvements of the SOD from the physicians’ perspective in a mixed-methods study, assessing the following research questions (RQ):

- RQ 1: What are the attitudes of physicians towards the concept of obtaining a second opinion and towards the SOD? (qualitative)
- RQ 2: What challenges and barriers are identified by physicians during the implementation phase of the SOD? (qualitative)
- RQ 3: How do physicians in Germany implement the SOD in daily practice? (quantitative)

## 2. Materials and Methods

This study is part of the ZWEIT-project [10], which examines the characteristics and the use of Second Opinion programs in Germany, as well as the needs and wishes from the perspectives of physicians and their patients, the potential service users. This study was supported by the Innovation Committee at the Federal Joint Committee (Innovation Fund, grant number 01VSF18014).

### 2.1. Study Design

**Figure 3:**
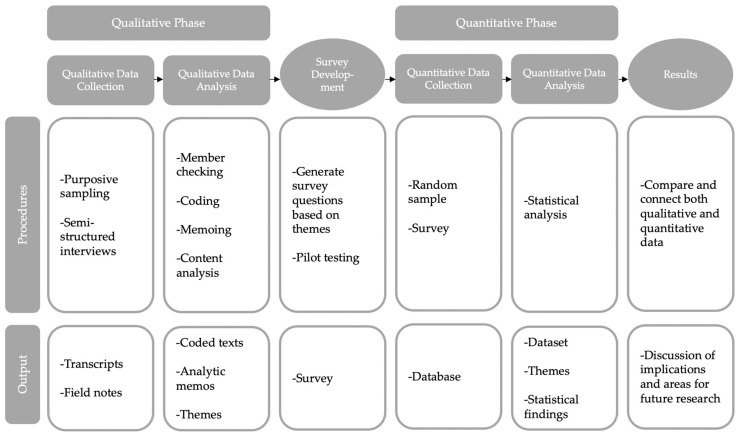
Study design diagram.

We followed an exploratory sequential mixed-methods design with an initial qualitative phase followed by a quantitative evaluation [11] (see Figure 3). A qualitative research methodology was used to explore why or how a given phenomenon occurs, in the present context to explore stakeholder-related experiences in the health care process, its reality, and to gain access to the object of the study. The subsequent quantitative follow-up survey was distributed to a larger group of physicians. This assisted in the quantitative measurement of the attitudes towards second opinion in general and the SOD, and provided impressions of the way the SOD is actually implemented in practice.

### 2.2. Recruitment

Interviews: We invited specialist physicians in gynecology and otorhinolaryngology, in line with the three types of surgery initially specified for the SOD procedure, namely hysterectomy, tonsillectomy, or tonsillotomy. Based on purposive sampling [12], experts to be interviewed were selected according to defined categories aiming to generate a heterogeneous sample with the broadest variety of different perspectives. The following categories were included: type of practice (primary physician-specialist vs. second opinion physician-specialist), region of practice (rural vs. urban) and main area of practice (outpatient vs. inpatient). For practical reasons, the interviews were limited to practitioners in the German states of Berlin and Brandenburg (3.7 and 2.5 million inhabitants, respectively). Eligible specialists who were already cooperating with the ZWEIT project were contacted by telephone. For detailed information on their role in the study, please see [9]. In keeping with common sample size standards for qualitative research [13], interviews were continued until we had achieved both of the following aims: collecting sufficient data to develop new knowledge about the study phenomenon, and proceeding to the point of generating no more new findings. The interviews were conducted by author SM between February and July 2020, initially in person, but from March 2020 onwards by telephone, owing to the COVID-19 pandemic.

Survey: The questionnaire was distributed by email. To reach our survey target, we created a database of 3305 e-mail addresses, which was based on publicly available information on otolaryngologists and gynecologists in Germany, provided by their respective professional associations. In addition, the professional associations were asked to forward the request to their members. After three weeks, a reminder email was sent to the entire sample. Data collection took place from 1 January through 31 March of 2021.

### 2.3. Data Collection Instruments

Interview Methodology: Guided, semi-structured interviews were conducted [14]. A preliminary interview guide was developed in a team including representatives of the disciplines of health sciences, psychology, and medicine (SM, DB, SvP), with further help from clinical experts (physician-specialists in gynecology and otorhinolaryngology), and further supplemented through an in-depth exchange with the specialists, and a close reading of the published theoretical literature about determinants of the successful implementation of innovations in the health care system, e.g., external context, professionals, and intervention [15]. The interview guide consisted of the following main topics: personal attitudes towards second opinion in general and specifically in relation to the SOD, as well as informants’ perspectives on the justifications for the implementation of the SOD, and obstacles encountered in the practical realization of the SOD requirements in everyday care (Supplementary Material File S1). In addition, we recorded socio-demographic data, gender, and profession. The preliminary interview procedure was piloted in three physician interviews, subsequently discussed, and adapted iteratively during the course of the project (SM). The interviews were recorded (SM) and transcribed verbatim; transcripts were pseudonymized in line with data-protection guidelines.

Survey development: Data from the qualitative study phase were used to develop a survey instrument for the subsequent quantitative phase of the study. In the first step, themes of the interviews were transformed into items for the survey (SM). After independent review and modification (DB), comments were discussed with the study team and adopted by consensus agreement (SM, DB, CR). In the third step, the questionnaire was pre-tested on three physicians and three external researchers who were not otherwise involved in the study; here, our objective was to refine wording and format, and to check whether predefined response options were exhaustive. Minor revisions were made accordingly. The survey addressed the following dimensions: awareness of the SOD, familiarity with the contents of the SOD, compliance in informing patients about the SOD, additional effort arising in daily practice, barriers affecting its implementation, attitudes towards second opinion in general and the SOD, and perceived need for adaptation of the SOD. We chose these themes because they are likely to have an impact on the implementation of the SOD. The derivation of the questionnaire items from the qualitatively identified themes are presented seen in Supplementary Material File S2. The survey included closed and open-ended questions.

### 2.4. Data Analysis

Qualitative Methods: Analyses were conducted by one researcher (SM) based on Kuckartz’s structured qualitative content analysis [16] using MAXQDA Analytics Pro 2022, Release 22.1.0, Verbi GmbH (Berlin, Germany). Categories were developed inductively to encompass the relevant material in the transcripts and memos using the data-driven development of a category system. Next, the category system was applied to the entire interview material. At this stage, data collection had already been completed. To ensure traceability, application of the category system was validated by an internal check whereby two researchers independently applied the developed category system to the entire material (SM, DB). Data collection and analysis were circular and continued until no substantially new findings emerged, indicating the attainment of theoretical saturation. We complied this manuscript in accordance with the Consolidated Criteria for Reporting Qualitative Research (COREQ) [17] (please see Supplementary Material File S3). For the presentation of the results, we selected representative quotes from the discussion transcript, which were included in this paper upon translation to English.

Quantitative Methods: The survey data were cleaned and analyzed descriptively using the SPSS Statistics for Windows, Version 23.0, IBM Corp: Armonk, NY, USA. The STROBE-checklist [18] was used to report quantitative data (please see Supplementary Material File S4). The procedure for conducting a detailed quantitative analysis is described in the Supplementary Material (please see Supplementary Material File S5).

Mixed-Methods: Here, we undertook building and merging integration techniques [12], where “building” refers to survey development, and “merging” involves the analytical integration of qualitative and quantitative data in joint displays. The GRAMMS reporting tool [19] was used to report mixed methods data (please see Supplementary Material File S6).

### 2.5. Ethical Considerations

This study was approved by the Ethics Committee of the Brandenburg Medical School (Theodor Fontane) (E-01-20190529). Potential participants in the qualitative research received a study information packet and provided their written informed consent prior to voluntary participation with pseudonymization. Participants in the quantitative study phase consented to the data collection by responding to the survey.

## 3. Results

### 3.1. Results—Qualitative Phase

A total of 22 specialist physicians were interviewed and their responses are included in the analysis. The characteristics of the interviewees are shown Supplementary Material File S7. The interviews lasted an average of 30 min. The category system is shown in Supplementary Material File S8.

#### 3.1.1. Physicians’ Perspectives on the Provision of Second Opinions as a Service in General

##### Patient-Related Aspects

Participating physicians viewed the opportunity for patients to obtain further information from a second opinion provider as a positive way of coping with their disease. In this regard, the physicians emphasized that patients can better accommodate their illness in a self-determined manner, while enjoying the full protection of their rights.

“*This is also primarily about patients’ rights. Every patient should have the right to seek a second opinion*.” (203Gyn, Pos. 2)

The opportunity to seek a second opinion promoted patient safety and, in particular, supported more confident decision making regarding disease management, through the provision of supplemental/additional information to the patient. Furthermore, respondents noted that unnecessary interventions might be avoided if patients sought a second opinion.

“*[...] a subjectively higher certainty. Let’s put it this way: if two people say the same thing, then the patient is more inclined to say: “Okay, that’s the way to go”. So, it’s a guarantee for the patient. And objectively, I would also say this avoids senseless medical measures*.” (211Gyn, Pos. 18)

In addition, the specialists mentioned that seeking a second opinion strengthens the patient–physician relationship.

#### 3.1.2. Profession-Related Aspects

Interviewees depicted positive aspects of SOD for their own medical practice, noting for example that indications could be made in a more reflective manner, and the information given to the patient was more comprehensible and precise.

“*That would probably also lead to reconsidering the indication. If you have to expect that the patient will ask again, I could imagine that the explanation could be simpler and more carefully [presented], I would say*.” (212Gyn, Pos. 389)

#### 3.1.3. Problems Arising When First and Second Opinions Differ

The physicians mentioned critical moments and challenges when seeking a second opinion. In particular, if the respective recommendations are divergent to their own, the patient remains faced with the unresolved problem of decision making, which may be accompanied by lingering uncertainty.

“*Well, the uncertainty. The uncertainty caused by the fact that two diverging opinions are expressed on one subject. And in the end, the patient cannot judge who is right*.” (210Oto, Pos. 36)

When first and second opinions differ, the relationship of trust may be affected, particularly for the physician who first recommended a surgery. The specialists emphasized that treatment could sometimes be delayed as a result of prolonging the decision-making process. This is particularly critical when concerning urgent medical examinations and procedures. Furthermore, from the physicians’ point of view, there is a risk that patients will switch to the second-opinion physician.

“*The risks I perceive are that if it’s about the surgery, the patient will be snatched away by other physicians. That a larger clinic with great equipment, with a great ambience, or with a particularly sympathetic surgeon, or whatever, might attract the patient. That you will lose the patient. There used to be a nice expression, “not coming back from the enemy flight” [in The German idiom means that you may prefer to remain in foreign territory]. That would be my fear*.” (212Gyn, Pos. 760)

#### 3.1.4. Attitudes towards the New German Second Opinion Directive (SOD)

In contrast to the topic of the general second opinion, aspects specifically concerning the SOD predominantly related to the medical routines of physicians rather than patient-related aspects. The response behavior with regard to self-related aspects of SOD appears particularly striking; a remarkable number of the interviewed physicians anticipated the needs of the patients.

#### 3.1.5. Structural Aspects Related to Successful Implementation in Daily Practice

The specialists addressed structural aspects that hinder the successful implementation of the SOD, including the deficient numbers of certified second-opinion physicians. The introduction of the SOD will likely exacerbate the well-known problem of the shortage of specialists. Especially in rural areas, patients likely have to travel long distances to consult an authorized second-opinion physician. Of note, physicians responded in the manner of advocating for patients, and sometimes did not inform them about the possibility of obtaining a second opinion.

“*I mean purely technically, in terms of implementation. My practice is in a rural area. And the nearest second physician is in R. [city], a good hundred kilometers away from here. It is completely utopian for me to send someone there for a second opinion*.” (218Gyn, Pos. 12)

For the physicians, the possibility of patients being able to book an appointment with a second-opinion physician ten days before the scheduled surgery seemed remote. The aspect of the ten-day limit once again highlights the shortage of qualified physicians for second opinion. At this point in our analysis, we considered whether the physicians interpreted the SOD correctly. For example, the SOD states that information about the right to a second opinion must be provided at least ten days prior to a scheduled surgery date. Some physicians incorrectly assumed that patients thus have only ten days to obtain a second opinion:

“*Well, it’s not practical because the patient only has ten days. So ultimately you have to go ten days before the surgery. And from my point of view, he/she won’t get a suitable appointment for a second opinion*.” (209Oto, Pos. 82)

The specialists reported an increased expenditure of organizational and bureaucratic efforts due to the information obligations specified in the SOD, which hampered their regular practice routines and increased the consultation time per patient.

“*So, it means that if I have someone [needing] a tonsillectomy, I now have to give them different documents. I have to obtain a signature to certify that I have informed them about the second opinion procedure. I have to file it again. I have to give them the list again for the second opinion physicians, which didn’t even exist until a year ago. That means my nurses copy it so that it can be given to the patients. That’s a lot of work. What I used to do in the past, telling [the patient] how it works and how to do it, now takes a lot more time. And that is simply a minus in the daily routine*.” (222Oto, Pos. 12)

#### 3.1.6. Defensiveness against the SOD

In some cases, respondents stated that the possibility for patients to receive a second opinion has always existed. Moreover, patients with a recommendation for surgery from an ambulatory practice have always been seen by a colleague in the surgical clinic, to double-check the appropriateness of the indication. Some interviewees consider the SOD as an unnecessary parallel effort to the existing dual-visit practice specific to the German health care system.

“*Second opinions have been standard anyway and the current procedure is highly complicated. The patients are highly dissatisfied*.” (213Gyn, Pos. 2)

Some physicians felt patronized by the introduction of the directive in relation to the practice of their profession. From their point of view, the SOD in effect questions their competence to provide adequate diagnoses and therapy recommendations.

“*That is a paternalism of our profession, of our performance. I find that quite unacceptable. This directive has to go in the trash bin*.” (215Gyn, Pos. 4)

A highly aversive attitude of some physicians was evident regarding the formal certification requirements for a second-opinion physician, which include Habilitation or an examiner certification, both of which are indicative of notable competence and a high status in the German health care system and academia. From the majority of the respondents’ point of view, there is no need for such requirements. Rather, they felt that all board-certified specialists should be allowed to provide a second opinion.

“*And now the question is why a declared second opinion process is needed. Why a second opinion specialist, who obviously first has to puff themselves up as a second opinion specialist in terms of their ego? Why should something better result now if we pre-specialize someone who can give the second opinion?*” (214Oto, Pos. 70)

#### 3.1.7. Criticism towards the Rationale behind the SOD

The physicians tended to criticize the justification of the SOD, which is primarily based on the so-called “Volume Criterion”. This states that SOD-eligible surgical procedures consist of those with increasing prevalence and/or strong regional variations, which may imply an unwanted expansion of surgical indications. From the interviewee’s point of view, the first tier of included surgeries (e.g., hysterectomies and removal of tonsils/tonsillar tissue) do not fulfil this primary criterion, because the respective numbers of these surgeries already decreased significantly, long before the directive was in place. Accordingly, the physicians did not accept the argument of increasing numbers of certain surgeries and questioned the rationale for the selection of indications.

“*After more than thirty years, we have gained a completely different perspective on these things [surgeries]. And that is why the number of hysterectomies is falling considerably. And that’s why this second opinion procedure no longer fits in with the times*.” (215Gyn, Pos. 38)

Moreover, participants emphasized that other types of surgery (and the associated indications for elective procedures) clearly do fulfil the Volume Criterion, but were nonetheless not selected in the first tier, and expressed that additional selection criteria beyond the Volume Criterion should be defined.

“*I see it very much from the patient’s point of view. And patients now ask for second opinions less frequently. So, this susceptibility to quantity is not at all of interest to them in the case of plannable, elective surgeries. But rather when it’s about something [serious]. So, when it is about tumour diseases, when it is about life-threatening diseases. When it comes to rare diseases, when it comes to major procedures in general*.” (210Oto, Pos. 14)

From a professional point of view, the elective surgeries studied here are preceded by other therapeutic interventions; only when all alternatives are exhausted is surgery presented as the last therapeutic option for the physician, which is conducive to the paternalistic attitudes of the physicians.

“*Before I indicate a hysterectomy, in most cases, unless there is a threat of something malignant or the symptoms are so severe, we have actually tried everything else. From a hormone coil to hormone therapy, and much more. Personally, HE is in last position for me*.” (212Gyn, Pos. 2011)

In addition, respondents noticed that patients take the prospect of undergoing surgery lightly, and should be willing take advantage of other treatment options before considering surgery.

“*[...] I’ve been in the business for a long time now and things have really changed in the last decade. The willingness of patients to undergo surgery has changed. Women are no longer willing to give up their uterus so lightly*.” (208Gyn, Pos. 26)

#### 3.1.8. Lack of Interest from Patients Who Want a Second Opinion

With regard to the use of a second opinion according to the SOD, the physicians stated that their patients were not greatly interested. In addition, from the point of view of the specialists interviewed, there is no general need for patients to seek a second opinion, neither for TE, nor in the case of HE:

“*So, if I now advise the surgery, I say: ‘Ok, you have the right to get a second opinion’. And then they say: ‘Oh no, I didn’t need it. Now it’s done and now it’s good’*.” (220Gyn, Pos. 36)

On the one hand, from the physicians’ point of view, the low use of second opinion inquiry highlighted by the directive, is explained by the time and effort patients have to invest in obtaining a second opinion.

“*So, most of them [patients] say, “I believe you,” and they don’t do it [seek a second opinion]. They have to go to someone who doesn’t work at the clinic where they are supposed to undergo surgery. I say in most cases it’s the children who are like that. Then it means another step for the parents—[who are] often working. And then finally to the clinic. And that is time-consuming for many people*.” (222Oto, Pos. 18)

On the other hand, the specialists alluded to regional characteristics and to the role of the patient–physician relationship:

“*To my patients who I want to undergo a hysterectomy, I of course offer this [obtaining a second opinion] according to the directive. Not a single one has taken up the offer and gone elsewhere. But that’s because we have few [...] walk-ins in this rural area. The ones you go after are those with a gynecologist around the corner*.” (Gyn212, Pos. 1199)

The qualitative results reviewed above were used to develop themes and questionnaire items for the survey (please refer to Supplementary Material File S2).

### 3.2. Results—Quantitative Phase

In total, from a survey of 3305 specialists, 146 questionnaires were returned, of which 10 were excluded from further analysis because less than half of the items were completed. Detailed sample characteristics are presented in Table 1

Overall, 71.1% (*n* = 136) of the surveyed physicians expressed a positive or a rather positive attitude towards second opinion in general. In contrast, only 32.4% of the specialists had a positive or a rather positive attitude towards the SOD, whereas the majority (67.6%) expressed a negative or a rather negative attitude. In particular, 61.8% of the respondents considered the SOD as superfluous, 22.1% considered it to be expandable, and only 16.2% deemed the SOD, in its current form, to be suitable. Detailed information as to why the SOD was often considered superfluous are shown in Table 2.

Almost all (95.6%) participants were aware of the SOD; 50% knew the content in detail; and 41.2% were broadly familiar with the content. Overall, 68.4% of the specialists reported informing patients about their rights in seeking a second opinion, 16.9% partly informed their patients, and 14.7% did not inform the patients. None of the physicians who reported informing their patients provided them with information exactly as intended in the SOD (for detailed information, see Supplementary Material File S9). Among those who do not inform their patients, the most frequently reported underlying reasons included the following: patients were not interested in seeking a second opinion; specialists did not consider the SOD to be relevant; and there was no broad implementation of SOD (for detailed information, see Supplementary Material File S10).

Regarding barriers for the successful implementation of the SOD, 77.9% of the specialists mentioned that performing the SOD in daily routine increased their workload. At least 36.8% stated that there were not enough approved second opinion physicians available, whereas 27.9% reported that they were unable to assess this availability. The response behaviour regarding the ten-day time limit was similar. In line with the findings about barriers, 31.6% of respondents mentioned that their patients experienced problems in securing an appointment ten days before surgery, while another third (31.6%) stated that there would be no such problems. Slightly more than half of the surveyed physicians reported that patients had no interest in seeking a second opinion, and almost half of the physicians stated that other indications were more relevant for patients.

The weighted participants’ responses are presented and combined with qualitative results in Table 3. The median values of variables for the corresponding hypotheses were significant and exceeded the means of the used scales as follows: “The SOD is not being implemented as intended (only items: decision making aids and others!)”, “The implementation of the SOD is connected with an additional effort in daily practice”, “Physicians have a rather positive attitude towards a second opinion in general”, “Physicians have a rather negative attitude towards SOD”, and “Physicians regard the SOD as superfluous”. There were no intersections between the confidence interval of the medians and the means of the used scale. Therefore, the hypotheses “The implementation of the SOD is connected with an additional effort in daily practice”, “Physicians have a rather positive attitude towards second opinion in general”, “Physicians have a rather negative attitude towards SOD”, and “Physicians regard the SOD as superfluous” were confirmed and the hypothesis “The SOD is not being implemented as intended” was confirmed only in part.

Compared to otolaryngologists, gynecologists were significantly less likely to have negative attitudes towards the SOD (Est = −0.56; *p* = 0.002) and to consider that the SOD demands additional effort in daily practice (Est = −0.40; *p* = 0.005). The gynecologists were also less likely to consider that other indications are more relevant for patients (Est = −0.34; *p* = 0.02). For both professional groups, there was a positive association between lack of knowledge about distance to the nearest certified second-opinion physician and positive attitudes towards second opinion in general (Est = 0.54; *p* = 0.01). Despite the lack of knowledge about distance to the nearest certified second-opinion physician, the respondents considered there to be a sufficient number of second-opinion physicians available (Est = 0.67; *p* = 0.001). On the other hand, the doctors lacking in knowledge about the distance to the nearest certified second opinion physician considered that SOD implementation brings an additional effort in daily practice (Est = 0.67; *p* = 0.001) and that certain other indications would be more relevant for patients (Est = 0.44; *p* = 0.01).

## 4. Discussion

This mixed-methods study explored the current state of the novel German Second Opinion Directive (SOD), which aims to provide guidance on structures and processes serving to reduce the number of potentially unnecessary surgical interventions and to improve medical decision making from a physician’s perspective, with focus on the recent implementation phase. Our interviews and survey findings indicate a generally positive professional attitude towards second opinions and towards their importance for the patient–physician relationship. At present, perceived inconsistencies and factual barriers interfere with the directive, collectively hampering its full acceptance and the general clinical implementation of the procedures as mandated by the current version of the directive. Based on the survey data, we formulated approaches to address such barriers.

Physicians often mentioned in the interviews that they were open in general terms to the principle of providing of second opinions in medical care in the present contexts of otolaryngology and gynecology. They emphasized the associated aspects of patient orientation, the benefits for supporting patients in the decision-making-process, and improved patient safety. The informants also highlighted their self-image as a medical professional in relation to the aim of providing the best possible care for patients, which reveals a positive attitude and a high level of patient orientation. Furthermore, the medical specialists emphasized that seeking a second opinion is everyday practice, thus calling into doubt the practical relevance and necessity of the SOD. A such, a negative attitude combined with self- and profession-related arguments emerged regarding the SOD. A tension between the second opinion as a general principle and the constraints of the SOD emerged clearly from this study. On the one hand, the physicians strived to perform in a patient-oriented way with an attitude that corresponds to the wishes, expectations, and satisfaction of the patients. On the other hand, the physicians emphasized structural challenges that prevent adequate implementation of the SOD [20].

We identified a limited acceptance of the SOD among our respondents, which may most plausibly be related to the lack of perceived relevance. The critical attitude of the physicians regarding the goal of reducing unnecessary surgeries must first be considered in light of the historically decreasing surgery rates of tonsillectomy and hysterectomy that long preceded the advent of the SOD [21,22,23]. Since the number of surgeries for the selected indications has demonstrably decreased in recent decades, there was less motivation to accept and implement the SOD in everyday care. From the physicians’ perspectives, economically induced volume increases are not to be expected for these indications. However, this attitude is at odds with the argument that the second opinion in general can contribute to increasing patient safety and the associated “avoidance of senseless medical measures”. One can assume that the acceptance rates of second-opinion programs will be lower when it concerns one’s own everyday practice. In this sense, we discerned a certain tension between the general and the specific aspects of the SOD. Obviously, our results show that an initially positive attitude towards the second opinion in general transforms into a negative perception when it effects a physician’s everyday practice by restricting their professional practice and autonomy through mandatory adherence to a directive. The impression of this tension based on the qualitative data was verified by the survey data. We conclude that the physician’s self-image of their everyday activities must be given greater consideration in the implementation of the SOD in order to guide the physicians in their everyday practice and thus promote a change in their routines.

For physicians, the requirement to inform patients about second-opinion options brings additional organizational work, arising from the need to provide patients with information material and to inform them about the possible benefits of second-opinion consultations. Under the present directive, physicians must regularly educate themselves about the current status and availability of approved second-opinion physicians. This increasing bureaucratization and formalization in the health care system seems to increase the workload of a physician in a way that reduces patient-oriented activities, possibly to the detriment of physician–patient relationships because of the unfavorable framework conditions and resultant decreases in patient consultation times [24].

From the perspective of the physicians, patients are confronted with the problem of finding a nearby authorized second-opinion physician, which imposes an additional burden on the patients. As a result, the physicians mentioned that seeking a second opinion according to the directive is relatively unpopular, and has been a rarity, especially in rural practices. In the context of a statement regarding the Healthcare Strengthening Act (The Healthcare Strengthening Act “Versorgungsstärkungsgesetz” was introduced to strengthen and ensure the provision of outpatient medical care) [25] the Marburger Bund (the largest German association of physicians) expressed concern as early as 2014 about the limited availability of authorized second-opinion physicians [26]. A recent annual hospital report on the developments in hospital financing in Germany also calls into question the practicality of obtaining a second opinion according to the SOD, especially in rural regions [27].

The underlying problem here derives from the high requirements for becoming an authorized second-opinion physician. The respondents in our study were critical of the stringent requirement for Habilitation or an examiner certification, which are markers of general, theoretical knowledge, but are not specifically relevant to the second opinion or to the resolution of the indication for surgery. The interviewed physicians viewed their medical status as being at risk from the new directives, and expressed that second opinion physicians should not be an elite specialization, since peers are sufficiently qualified and competent to provide a reliable second opinion. Accordingly, in our view, the criteria could be made less stringent, so as to facilitate the sufficient access of patients to authorized second-opinion physicians; this is especially important for patients living in rural areas, who are otherwise disadvantaged compared to their urban counterparts. The results of a recent study support these findings [28].

Physicians often interpreted the SOD as providing patients with only ten days to receive a second opinion after being informed of their right to seek one, whereas the SOD actually states that there must be a period of at least ten days between receiving information about seeking a second opinion and the planned surgery. Obviously, the ten-day period is misinterpreted by physicians, which calls for an unambiguous rewording of the language in the SOD.

Besides structural challenges that hamper efficient and goal-oriented implementation in daily practice, our interviews also revealed a more generic restraint towards the SOD. One likely reason for this lies in the range of available options for seeking a second opinion in the German health care system. When examined in the context of underuse and misuse of health care services, the implementation of the SOD seemingly leads to redundancy in the available health care structures. Second, the choice of eligible surgical indications was questioned by our interviewees. The first version of the SOD, as investigated in this study, was restricted to only three non-oncological surgical indications in gynecology and otolaryngology, all of which experienced a substantial decline in frequency in recent years owing to other developments that have served to reduce unnecessary medical interventions in these domains [21,22,23]. Of note, the interviewees were professionals in the two aforementioned fields, whose views, experiences, and opinions regarding these circumscribed health care settings may not be representative of those of other medical professionals. In the meantime, the SOD has undergone several iterations, and currently includes seven indications (please refer to Supplementary Material File S11). A new survey of other specialists, e.g., orthopedists, with indications that have not decreased numerically, might yield differing results.

We demonstrated that the SOD is not yet implemented by physicians in Germany in the manner intended. According to our data, for various reasons, physicians do not provide all the relevant information stipulated when explaining the prospects and rights for obtaining a second opinion to their patients. Physicians are not familiar with the detailed content of the SOD, as confirmed by another recent survey among 2764 medical professionals in Germany [28]. These findings indicate a need for better communication of the SOD’s contents to physicians. Presumably, the individual physician’s attitude towards the SOD plays a significant role in determining the provision of second opinions when providing patients with information. Beyond the structural issues and the more generalized, plausible restraint mentioned above, a further contributing factor to incomplete implementation might be that physicians are more disposed to accept guidelines developed by their own professional organization than those imposed by governmental authorities [29]. Obviously, emphasizing physician participation in the development of Directives and increasing the awareness for the regularization of Directives, e.g., via professional societies, remain important issues for the improvement and future sustainment of physicians’ confidence in the SOD.

We consider our study design to be highly applicable to the research topic and questions, insofar as we directly assessed the physicians’ perspectives and then proceeded to confirm our findings with quantitative data. However, not all qualitatively identified topics could be verified in this study. While this might constitute a limitation, the disparate results also underline the complexity of the SOD. Furthermore, our study design was appropriate given that the object of research is still sparsely investigated due to its recent onset. The mixed-methods study design has the advantage that the perceived challenges can be described in detail, based first on the experiences of the participants as conveyed through a structured interview, with follow-up quantitative validation of the qualitative findings. The qualitative research phase provided an in-depth understanding of the impact of the SOD on primary healthcare in Germany. Due to the open and exploratory approach, interview partners were able to provide thorough narrative accounts of their experience. We concede certain limitations exist arising from our data collection in only one region of Germany. This may limit the generalizability of the findings owing to local referral physician shortages, which may have adversely affected attitudes towards the SOD. The recruitment of participants was conducted during the outbreak of the COVID-19 pandemic, which necessitated extraordinary re-organization and resulted in staff shortages. Only physicians who cooperated with the ZWEIT study participated in the qualitative phase, and only two certified second-opinion physicians were recruited for the interviews. The results may therefore be vulnerable to selection bias. With regard to the questionnaire survey, the geographic catchment area was larger, although the participation rate was exceedingly low (<1%). It is possible that strongly negative appraisals of the SOD contributed to selection bias. In addition, the representativeness of the sample cannot be ensured due to the limited number of participants. Furthermore, the survey has not been validated yet and the results for only three procedures may not be transferable to the broadening scope of SOD.

Our present research focused on possible barriers for physicians to implement the SOD in its initial phase. Further research is needed to assess the perception of physicians regarding their use of the SOD, whether the current Directive is being implemented nationwide in Germany, and whether regional differences exist, especially between rural and urban regions. Furthermore, the suitability of current indications in the SOD remains to be established. Newly added indications in the directive, particularly shoulder arthroscopy and the implantation of knee prothesis, entail much higher volumes of use (refer to Supplementary Material File S12 for surgery numbers in the years 2005 to 2020) [30]. We predict that differences will emerge in the physician information provision practices concerning more common surgeries. Furthermore, the results of the qualitative data should be validated using a larger sample. We identified a need for the reassessment of the stringent authorization criteria for second-opinion physicians. Thus, the focus of considerations should not be confined to the implementation of the directive by physicians, but should include the patient perspective, a matter to be explored in the SOD evaluation commissioned by the Federal Joint Committee [31].

## 5. Conclusions

Gynaecology and Otolaryngology physicians in Germany support the concept of seeking a second opinion. However, they express a negative attitude towards the SOD, which they tend to regard as superfluous. Among the chief obstacles to its implementation were the additional effort it entailed in daily practice, the limited number of available authorized second-opinion physicians due to the stringent requirement, and lingering questions about the choice of eligible surgical indications. Future development of the SOD needs to be better focused and aligned with everyday practice, and should give greater consideration to the patient’s perspective.

## Figures and Tables

**Table 1:** Characteristics of 136 participants in the survey.

Characteristics	Participants (*n* = 136)	
	*n*	%
Professional group		
Otolaryngology	59	43.4%
Gynaecology	77	56.6%
Population density (No. of inhabitants)		
<10,000	8	5.9%
10,000 < 100,000	68	50.0%
≥100,000	59	43.4%
missing	1	0.7%
Years of experience as specialist physician		
<6 years	3	2.2%
6–10 years	13	9.6%
11–15 years	18	13.2%
≥16 years	100	73.5%
missing	2	2.7%
Type of Practice		
Single practice	72	52.9%
Joint practice with shared health insurance license	40	29.4%
Joint practice with separate health insurance license	5	3.7%
Medical centre (MVZ)	6	4.4%
Hospital	7	9.5%
Hospital and single practice	1	1.3%
Currently no professional practice	3	4.0%
Missing	2	2.7%
Distance to the nearest certified second opinion physician		
Less than 25 km away	62	45.6%
More than 25 km away	32	23.5%
I don’t know	42	30.9%
Age		
<45 years	20	14.7%
≥45 years	115	84.6%
Missing	1	0.7%

**Table 2:** Results of the ZWEIT Physician Questionnaire Survey Study among Otolaryngology and Gynaecology Practitioners in Germany (2021).

Items	Participants (*n* = 136)	
	*n*	%
Awareness of the second opinion directive		
Yes	130	95.6%
No	6	4.4%
Familiarity with the contents of the second opinion directive		
I am familiar with the contents in detail	68	50.0%
I am broadly familiar with the contents	56	41.2%
I don’t know the contents	6	4.4%
Missing	6	4.4%
Informing patients about the second opinion directive for the surgery indications TE/TT or HE ^1,2^		
yes	93	68.4%
no	20	14.7%
partly	23	16.9%
Additional effort in daily practice		
yes	106	77.9%
no	19	14%
cannot assess	11	8.1%
Sufficient number of authorised second opinion physicians available		
Yes	50	36.8%
no	48	35.3%
cannot assess	38	27.9%
Patients can assess a second opinion appointment about 10 days to surgery		
yes	43	31.6%
no	43	31.6%
cannot assess	50	36.8%
Patients are interested in obtaining a second opinion		
yes	49	36%
no	69	50.7%
cannot assess	18	13.2%
Other indications are more relevant for patients		
yes	60	44.1%
no	28	20.6%
cannot assess	48	35.3%
Attitude towards second opinion in general		
positive	39	28.7%
rather positive	59	43.4%
rather negative	23	16.9%
negative	15	11%
Attitude towards second opinion directive		
positive	10	7.4%
rather positive	34	25.0%
rather negative	63	46.3%
negative	29	21.3%
I consider the SOD to be		
rather suitable	22	16.1%
rather expandable	30	22.1%
rather superfluous	84	61.8%
Potential ways to adapt the second opinion directive suitable ^3^		
Increase the number of authorised second opinion physicians	47	34.5%
Adapt the authorisation requirements to become a second opinion physician	50	36.7%
Extend the 10-day limit	62	45.5%
Improve availability of information on second opinion providers	70	51.4%
Others	47	34.5%

^1^ Since the SOD does not cover surgeries *per se*, but rather specific elective indications for surgery, both the SOD and this ZWEIT sub-study only include patients/clinical situations in which the indication for surgery is unrelated to (suspected) cancerous growths. ^2^ See Supplementary Material Files S9 and S10 for more detailed results on the subgroups who report informing (*n* = 93) or not informing (*n* = 20) their patients about the SOD. ^3^ Multiple responses possible.

**Table 3:** Joint Display: Qualitative Data and Descriptive statistic of weighted participants’ responses.

Theme	Qualitative Interviews Data-Driven Hypothesis	Item Questionnaire		*n*	Formal Scale Mean	Mean (SD)	Median (95%CI)
Attitudes towards second opinion in general	Physicians have a rather positive attitude towards second opinion in general.	What is your basic attitude towards second opinion in general?		136	**2**	2.38 (1.04)	**2.25 (2.25; 3.0)**
Attitudes towards SOD	Physicians have a rather negative attitude towards SOD.	What is your basic attitude towards the SOD?		136	**2**	2.36 (1.05)	**2.25 (2.25; 3.0)**
The SOD is superfluous	Physicians regard the SOD as superfluous.	I consider the SOD to be rather suitable, rather expandable or rather superfluous.		136	**1.5**	2 (0.93)	**2.25 (2.0; 2.25)**
Reasons for inadequate implementation	The Selection of indications is inadequate.	In your opinion, do you think other indications—than those mentioned in the SOD—are more relevant for patients?		136	1.5	1.85 (0.85)	2 (1.5; 2.0)
Reasons for inadequate implementation	The implementation of the SOD is connected with an additional effort in daily practice.	Does the current SOD lead to additional organisational work (e.g., interruptions in the practice routine or increased documentation requirements)?		136	**1.5**	2.21 (0.89)	**2.25 (2.25; 3.0)**
Reasons for inadequate implementation	The number of available authorised second opinion physicians is insufficient.	In your opinion, are there sufficient approved second opinion specialists available to patients in your area?		136	1.5	1.66 (0.89)	1.5 (1.5; 2.0)
Reasons for inadequate implementation	Patients are not interested in seeking a second opinion.	Do you feel that patients are open to the theme of obtaining a second opinion?		136	1.5	1.77 (0.95)	2 (1.5;2.25)
Implementation of the SOD	The SOD is not being implemented as intended.	When explaining the patients to have the right seeking a second opinion, which aspects do you include?	Information services about certified second opinion physicians	93	0.5	0.46 (0.45)	0.75 (0; 0.75)
			Decision-making tool of the IQWIG	93	**0.5**	0.71 (0.37)	**0.75 (0.75; 1.00)**
			Information about the release of the medical report	93	0.5	0.54 (0.44)	0.75 (0.5; 0.75)
			Distribution of the patient information sheet	93	0.5	0.22 (0.39)	0 (0;0)
			Others	93	**0.5**	0.78 (0.33)	**1 (0.75;1.00)**

## Data Availability

All data relevant to the study are included in the article. For further questions regarding the reuse of data, please contact the corresponding author (susann.may@mhb-fontane.de).

## Supplementary Materials

The following supporting information can be downloaded at: https://www.mdpi.com/article/10.3390/ijerph19127426/s1, Supplementary Material File S1: Interview Guide; Supplementary Material File S2: Joint display; Supplementary Material File S3: COREQ; Supplementary Material File S4: STROBE, Supplementary Material File S5: Depiction detailed quantitative analysis [32,33,34,35,36]; Supplementary Material File S6: GRAMMS; Supplementary Material File S7: Characteristics of the participants qualitative; Supplementary Material File S8: Category system; Supplementary Material File S9: Results of subgroup 1; Supplementary Material File S10: Results of subgroup 2; Supplementary Material File S11: History of the admission of indications into the SOD; Supplementary Material File S12: Number of inpatient surgeries.
