# Supplementary material for: Physicians’ Perspectives on the Implementation of the Second Opinion Directive in Germany—An Exploratory Sequential Mixed-Methods Study
Source: Int J Environ Res Public Health. 2022 Jun 17;19(12):7426. doi: 10.3390/ijerph19127426 (PMC9224158; doi:10.3390/ijerph19127426)
Supplement: Supplementary file 1 [file ijerph-19-07426-s001.zip › Supplementary Material File S10_Results of the subgroup 2.pdf]

Supplementary Material File S10

Table S5. Results of the subgroup: physicians, who not inform patients about their right to seek a second opinion under the SOD (n=20)

**Physicians who report NOT informing their eligible patients about the right to obtain a second opinion under the SOD**	**Participants (n = 20)**	
	**n**	**%**
Are unaware of the SOD	2	10
Rejection of the SOD	2	10
Patients are not interested	9	45
Forget about providing SID information because the indication is rarely given	4	20
SOD is not meaningful	9	45
No benefits for me from following the SOD	5	25
No obvious consequences to physician if SOD information is not given	2	10
The SOD is not implemented very much overall	7	35
Other	6	30
