# Supplementary material for: Physicians’ Perspectives on the Implementation of the Second Opinion Directive in Germany—An Exploratory Sequential Mixed-Methods Study
Source: Int J Environ Res Public Health. 2022 Jun 17;19(12):7426. doi: 10.3390/ijerph19127426 (PMC9224158; doi:10.3390/ijerph19127426)
Supplement: Supplementary file 1 [file ijerph-19-07426-s001.zip › Supplementary Material File S11_History of the admission of indications into the SOD.pdf]

Supplementary Material File S11

Table S6. History of the admission of indications into the SOD*

Indication	Implementation date
Hysterectomy, tonsillotomy, tonsillectomy	8.12.2018
Shoulder arthroscopy	20.02.2020
Implantation of knee endoprosthesis	12.01.2021
Amputation for diabetic foot syndrome	27.05.2021
Spinal surgery	19.11.2021

*planned procedures: catheter-based electrophysiological cardiac examinations and cardiac ablation
