# Supplementary material for: Physicians’ Perspectives on the Implementation of the Second Opinion Directive in Germany—An Exploratory Sequential Mixed-Methods Study
Source: Int J Environ Res Public Health. 2022 Jun 17;19(12):7426. doi: 10.3390/ijerph19127426 (PMC9224158; doi:10.3390/ijerph19127426)
Supplement: Supplementary file 1 [file ijerph-19-07426-s001.zip › Supplementary Material File S12_Number of inpatient surgeries.pdf]

Supplementary Material File S12

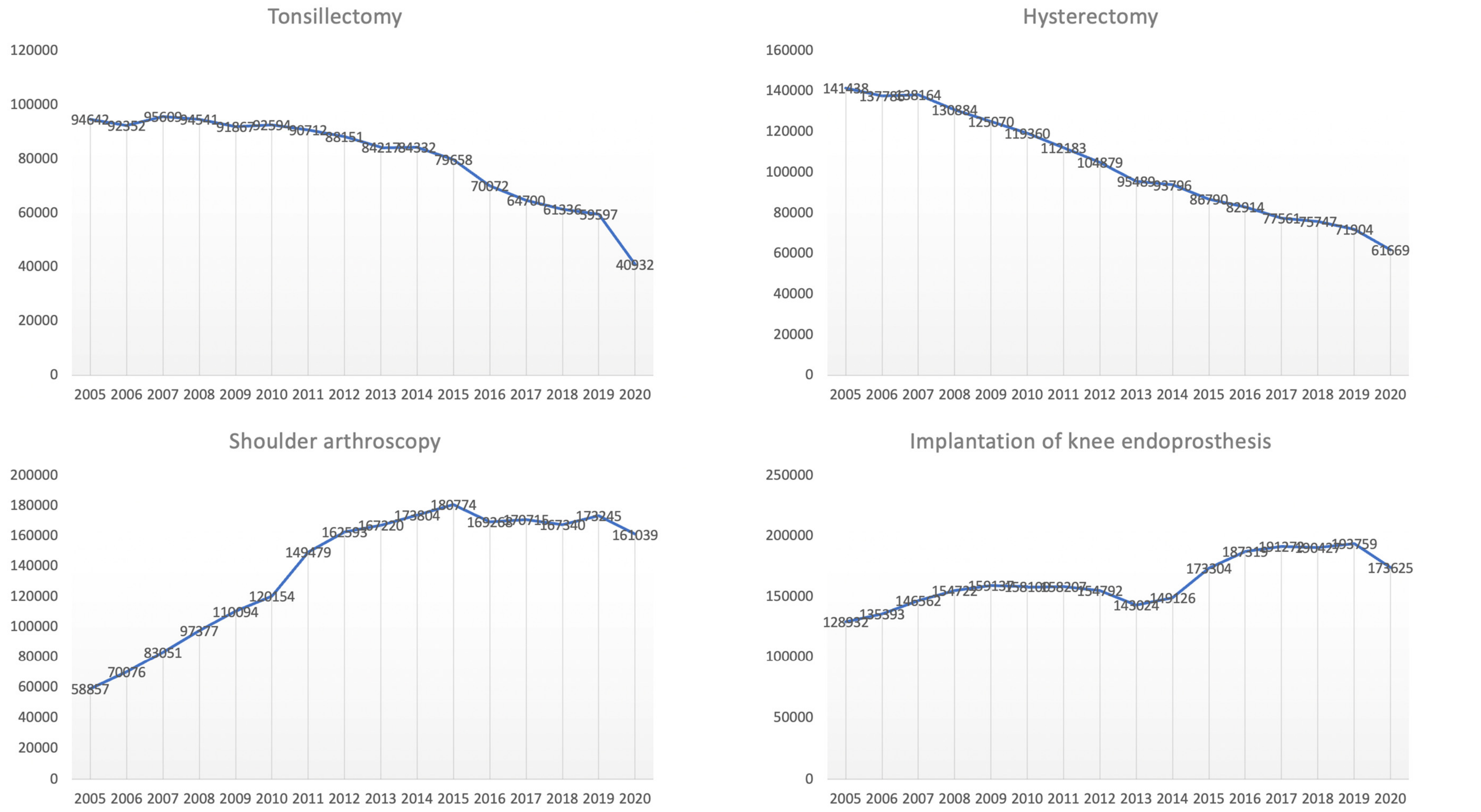

Figure S2: Number of inpatient surgeries from 2005 and 2020 - data provided by Federal Statistical Office of Germany [2022]; own illustration
