# Supplementary material for: Physicians’ Perspectives on the Implementation of the Second Opinion Directive in Germany—An Exploratory Sequential Mixed-Methods Study
Source: Int J Environ Res Public Health. 2022 Jun 17;19(12):7426. doi: 10.3390/ijerph19127426 (PMC9224158; doi:10.3390/ijerph19127426)
Supplement: Supplementary file 1 [file ijerph-19-07426-s001.zip › Supplementary Material File S1_Interview guide.pdf]

Supplementary Material File S1

Table S1. Interview guide specialists ZWEIT

Guiding questions	Check aspects
What do you think about second opinions in general? What`s your perspective on this?<br><br>In which situations do you think it is helpful for patients to seek a second opinion? (e.g., indications, medical history, diagnostics, treatment)<br><br>What options for seeking a second opinion do you perceive?<br><br>What risks do you perceive in regard of seeking a second opinion in general?<br><br>How does seeking a second opinion change the physician-patient-relationship?<br><br>Second opinions are supposed to support the patient in decision-making. What is your position on this?<br><br>Second opinions are supposed to avoid medically unnecessary surgeries. How do you perceive this?	Attitudes towards second opinion in general<br><br>Opportunities and risks of seeking a second opinion in general
What are your experiences as a physician with the new directive on the second opinion procedure?<br><br>What do you think about SOD? What`s your perspective on this directive?<br><br>Do you perceive any new opportunities in regard to (improved?) SOD implementation? If so, please explain.<br><br>Do you perceive any problems or risks regarding SOD implementation? Did you identify any particular difficulties? If so, where?<br><br>How does SOD influence your daily practice? Has the implementation of SOD changed anything in your daily practice?<br><br>How do you inform patients about their right to a obtain a second opinion? When informing patients about their right to seek a second opinion, what aspects do you include in the discourse?<br><br>How do patients react when inform them about their right to seek a second opinion?	Attitudes towards SOD<br><br>Opportunities and risks of SOD<br><br>Impact on everyday practice<br><br>Experiences with the SOD<br><br>(Note: spatial distance, time feasibility)
