# Supplementary material for: Physicians’ Perspectives on the Implementation of the Second Opinion Directive in Germany—An Exploratory Sequential Mixed-Methods Study
Source: Int J Environ Res Public Health. 2022 Jun 17;19(12):7426. doi: 10.3390/ijerph19127426 (PMC9224158; doi:10.3390/ijerph19127426)
Supplement: Supplementary file 1 [file ijerph-19-07426-s001.zip › Supplementary Material File S2_Joint Display.pdf]

Supplementary Material File S2

Table S2. Joint display mapping qualitative themes and categories to the developed questionnaire (integration of qualitative & quantitative phase)

**Qualitative theme and category**	**Questionnaire Items**
Awareness of the Second Opinion Directive (SOD)	
Some physicians are not aware of the SOD.	Are you aware that there is a SOD for certain indications?
The physicians are not familiar with the contents of the SOD.	Are you familiar with the contents of the SOD
Informing about seeking a second opinion	
Some physicians do not inform the patients about the right to obtain a second opinion.	Do you inform your patients about their right to seek a second opinion for the indications TE/TT and HE?
The information about seeking a second opinion is not provided as intended in the SOD.	When explaining the patients to have the right seeking a second opinion, which aspects do you include?
Physicians provide information about seeking a second opinion if patients are interested.	Do you always distribute the offers?
The information is not provided because doctors are negatively disposed towards the SOD.	Why don't you inform patients about their right to seek a second opinion?
Patients are not interested in seeking a second opinion.	Do you feel that patients are open to the concept of obtaining a second opinion?
Challenges and barriers	
The SOD leads to an additional effort in daily practice.	Does the current SOD lead to additional organisational work (e.g., interruptions in the practice routine or increased documentation requirements)?
The number of certified SOD physicians is insufficient.	In your opinion, are there sufficient approved second opinion specialists available to patients in your geographic area?
Patients do not get an appointment with a second opinion physician within ten days before surgery.	In your opinion, would patients be able to get an appointment with an authorised second

	opinion specialist within ten days before the planned surgery?
The selection of indications is inadequate.	In your opinion, do you think that other indications than those mentioned in the current SOD are more relevant for patients?
Attitudes towards second opinion in general and towards the SOD	
Physicians have a rather positive attitude towards second opinion in general.	What is your basic attitude towards second opinion in general?
Physicians have a rather negative attitude towards SOD.	What is your basic attitude towards the SOD?
Physicians consider the SOD is superfluous.	I consider the SOD to be…<br>… rather suitable<br>… rather expandable<br>… rather superfluous
The SOD should be adapted.	Which aspects of the Second Opinion Directive would need to be adapted from your point of view?
