# Supplementary material for: Physicians’ Perspectives on the Implementation of the Second Opinion Directive in Germany—An Exploratory Sequential Mixed-Methods Study
Source: Int J Environ Res Public Health. 2022 Jun 17;19(12):7426. doi: 10.3390/ijerph19127426 (PMC9224158; doi:10.3390/ijerph19127426)
Supplement: Supplementary file 1 [file ijerph-19-07426-s001.zip › Supplementary Material File S5 Depiction detailed quantitative analysis.pdf]

Depiction detailed quantitative analysis

The quantitative analysis was conducted in four steps, as follows. In the first step, participants' attitudes to SODs as reported in the survey were recoded from left to right according the direction of the hypotheses, e.g., the response options in relation on the *a priori* hypothesis that "Physicians have a rather positive attitude towards second opinion in general", which ranged from the negative to the positive scale. The answers "negative", "rather negative", "rather positive" and "positive" were coded as "1", "2", "3" and 4, respectively. The undefined "cannot access" response option was coded with an intermediate value, e.g., the options "no", "cannot access", "yes", were encoded by the values "1", "2", "3", respectively. The same incremental coding was undertaken for the characteristics indicated by the participants, e.g., for the characteristic "Population density", numbers of inhabitants of <10.000, 10.000, <100.000, and ≥100.000 were coded as "1", "2", "3", "4", respectively. Categorical characteristics were encoded dichotomously as 1 and 0: Professional group (Gynecology vs. Otolaryngology), Type of Practice (Single practice vs. others), Distance to the nearest certified second opinion physician (less than 25 km and more than 25 km away vs. "I don't know"). The second step in our quantitative analysis involved processing the data to conduct an optimally sensitivity analysis [1]. The participants' familiarity with the contents of the SOD was used to develop a weighting of their attitude ratings. Each degree of familiarity was assigned its own weight: "I am familiar with the contents of SOD in detail" = 1, "I am broadly familiar with the contents of SOD" = 0.75, "I don't know the contents of SOD" = 0, Missing = 0.5. All reported participants' attitudes were then refined by multiplying by these weightings. Results at this stage were interpreted as constituting a sensitivity analysis of exploratory character. In the third step, we checked the distribution of weighted participants' responses. The examined hypothesis was assumed to be confirmed when 1) the median value of responses was greater than the formal mean of the used scale, and 2) there were no intersections between the confidence interval of the median and the formal mean of the used scale. In order to assess the significance of the median scores, we calculated two-sided 95% bootstrap confidence intervals based on 1000 bootstrap replications using the ci_median function () of the R-software package 'confintr' (Mayer M. (2022) confintr: R package version 0.1.2). If the 95% confidence interval did not include the null value, we could conclude that there was a statistically significant difference between observed and simulated groups ($p<0.05$) [2]. Finally, in the fourth step, we undertook an exploratory examination of the associations between participants' characteristic and attitudes to SOD. Since we expected the data to contain deviations from the normal distribution, we used robust statistics. For this analysis, we applied a number of multiple linear robust regressions using the lmrob () function of the R-package 'robustbase' [3]. This function computes an MM-type regression estimator as described in Yohai [4] and Koller and Stahel [5]. By default, the regression uses a bi-square redescending score function, and it returns a highly robust and highly efficient estimator (with 50% breakdown point and 95% asymptotic efficiency for normal errors). For the robust regression analysis, the participants' demographic characteristica and attitudes to SOD were assumed to be dependent variables. The independent variables were Age, Professional group, Population density, Years of experience as specialist physician, Type of Practice, and Distance to the nearest certified second opinion physician. All analyses with R-packages were run version 1.4.1103 (R Core Team, 2021).

Reference

1. Saltelli A, Chan K, Scott M. Sensitivity Analysis: Gauging the Worth of Scientific Models. New York: John Wiley & Sons (2000).
2. Cox D.R., Hinkley D.V. (1974) Theoretical Statistics, Chapman & Hall.
3. Maechler M, Rousseeuw P, Croux C, Todorov V, Ruckstuhl A, Salibian-Barrera M, Verbeke T, Koller M, Conceicao EL, Anna di Palma M (2021). robustbase: Basic Robust Statistics. R package version 0.93-9, http://robustbase.r-forge.r-project.org/.

4. Yohai, V. J. (1987). High breakdown-point and high efficiency robust estimates for regression. The Annals of statistics, 642-656.
5. Koller, M., & Stahel, W. A. (2011). Sharpening wald-type inference in robust regression for small samples. Computation-al Statistics & Data Analysis, 55(8), 2504-2515.
