# Supplementary material for: Physicians’ Perspectives on the Implementation of the Second Opinion Directive in Germany—An Exploratory Sequential Mixed-Methods Study
Source: Int J Environ Res Public Health. 2022 Jun 17;19(12):7426. doi: 10.3390/ijerph19127426 (PMC9224158; doi:10.3390/ijerph19127426)
Supplement: Supplementary file 1 [file ijerph-19-07426-s001.zip › Supplementary Material File S6_GRAMMS.pdf]

Good Reporting of a Mixed Methods Study (GRAMMS) checklist

Guideline	Section: page
Describe the justification for using a mixed methods approach to the research question	Study design: 4
Describe the design in terms of the purpose, priority and sequence of methods	Methods: 4-6
Describe each method in terms of sampling, data collection and analysis	Methods: 4-6
Describe where integration has occurred, how it has occurred and who has participated in it	Results - Mixed methods findings: 12-16
Describe any limitation of one method associated with the present of the other method	Strengths and limitations: 19-20
Describe any insights gained from mixing or integrating methods	Results - Mixed methods findings: 12-16

O'Cathain A, Murphy E, Nicholl J. The quality of mixed methods studies in health services research. J Health Serv Res Policy. 2008;13: 92-98.
