# Supplementary material for: Physicians’ Perspectives on the Implementation of the Second Opinion Directive in Germany—An Exploratory Sequential Mixed-Methods Study
Source: Int J Environ Res Public Health. 2022 Jun 17;19(12):7426. doi: 10.3390/ijerph19127426 (PMC9224158; doi:10.3390/ijerph19127426)
Supplement: Supplementary file 1 [file ijerph-19-07426-s001.zip › Supplementary Material File S7_Characteristics of participants qualitative.pdf]

## Supplementary Material File S7

Table S3: Characteristics of participants in the ZWEIT Physician Interview Study

| ID | Specialist | Setting | Physician | Gender |
|---|---|---|---|---|
| 201Gyn | Gynaecology | Outpatient | Primary physician | Female |
| 202Oto | Otolaryngology | Outpatient | Primary physician | Female |
| 203Gyn | Gynecology | Outpatient | Primary physician | Male |
| 204Gyn | Gynecology | Outpatient | Second opinion physician | Female |
| 205Oto | Otolaryngology | Outpatient | Primary physician | Female |
| 206Oto | Otolaryngology | Outpatient | Primary physician | Female |
| 207Oto | Otolaryngology | Outpatient | Primary physician | Male |
| 208Gyn | Gynecology | Outpatient | Primary physician | Female |
| 209Oto | Otolaryngology | Outpatient | Primary physician | Male |
| 210Oto | Otolaryngology | Inpatient | Primary physician | Male |
| 211Gyn | Gynecology | Outpatient | Second opinion physician | Male |
| 212Gyn | Gynecology | Outpatient | Primary physician | Male |
| 213Gyn | Gynecology | Inpatient | Primary physician | Male |
| 214Oto | Otolaryngology | Outpatient | Primary physician | Male |
| 215Gyn | Gynecology | Outpatient | Primary physician | Male |
| 216Oto | Otolaryngology | Outpatient | Primary physician | Male |
| 217Oto | Otolaryngology | Outpatient | Primary physician | Female |
| 218Gyn | Gynecology | Outpatient | Primary physician | Female |
| 219Gyn | Gynecology | Outpatient | Primary physician | Male |
| 220Gyn | Gynecology | Outpatient | Primary physician | Female |
| 221Gyn | Gynecology | Outpatient | Primary physician | Male |
| 222Oto | Otolaryngology | Outpatient | Primary physician | Female |
