# Supplementary material for: Physicians’ Perspectives on the Implementation of the Second Opinion Directive in Germany—An Exploratory Sequential Mixed-Methods Study
Source: Int J Environ Res Public Health. 2022 Jun 17;19(12):7426. doi: 10.3390/ijerph19127426 (PMC9224158; doi:10.3390/ijerph19127426)
Supplement: Supplementary file 1 [file ijerph-19-07426-s001.zip › Supplementary Material File S9_Results of the subgroup 1.pdf]

Supplementary Material File S9

Table S4. Results of the subgroup: physicians, who inform patients about the right seeking a second opinion (n=93)

Physicians who report informing their patients about the right to obtain an SOD	Participants (n = 93)	
	n	%
Aspects of information exchange		
Information services about certified second opinion physicians	43	46.2
Decision-making tool of the IQWIG[1]	17	18.2
Information about the release of the medical report	69	74.1
Distribution of the patient information sheet	7	7.5
Others	11	11.8
Do you always disseminate the second opinion offers?		
Yes	33	35.5
No, only if the patients are interested	59	63.5
Missing	1	1.0

[1] IQWIG: https://www.gesundheitsinformation.de/zweitmeinung-vor-operationen.html
