# Supplementary figures and images for: Physicians’ Perspectives on the Implementation of the Second Opinion Directive in Germany—An Exploratory Sequential Mixed-Methods Study
Source: Int J Environ Res Public Health. 2022 Jun 17;19(12):7426. doi: 10.3390/ijerph19127426 (PMC9224158; doi:10.3390/ijerph19127426)

Supplementary Material File S8

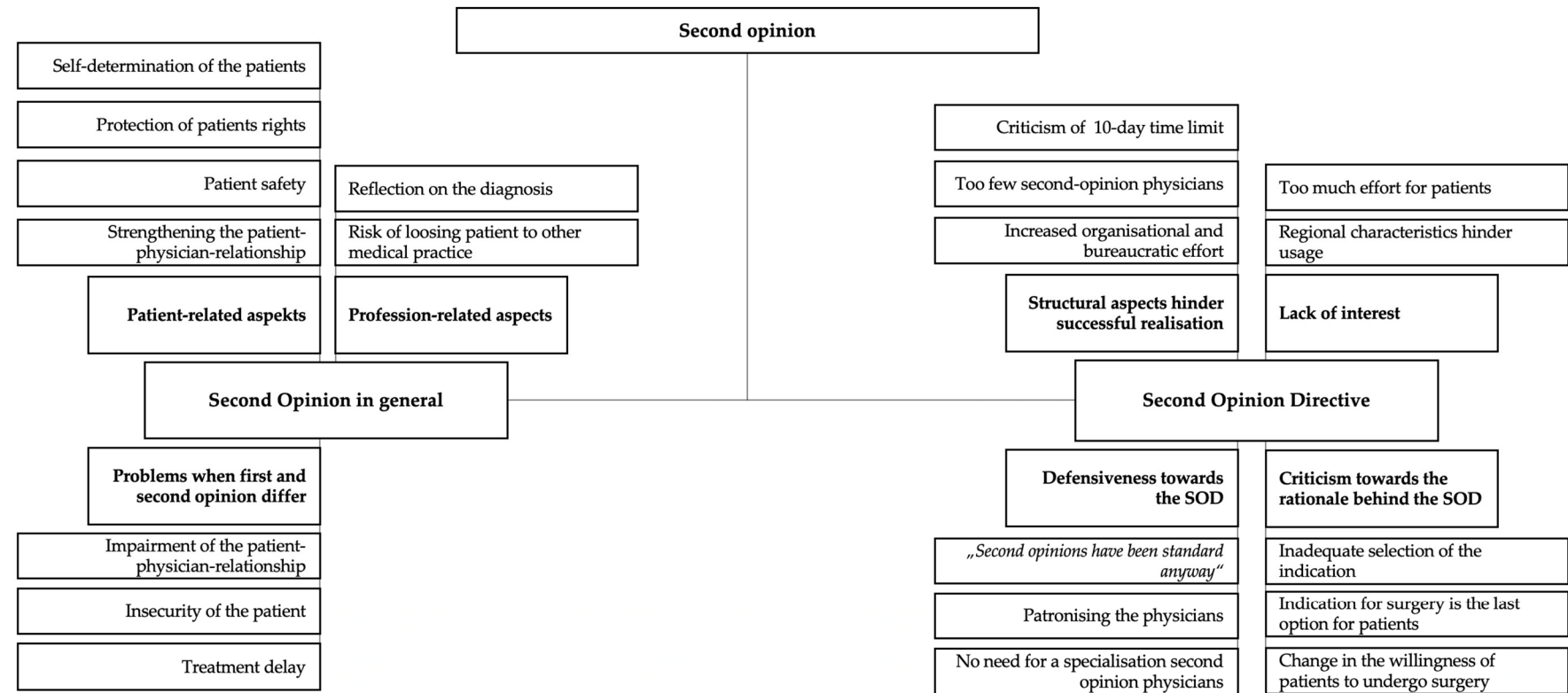

Figure S1: Category system

Supplement: Supplementary file 1 [file ijerph-19-07426-s001.zip › Supplementary Material File S8_Category system.pdf]
